# Preservation effect of plant essential oil-KGM composite coating materials on the tomatoes

**DOI:** 10.1371/journal.pone.0328192

**Published:** 2025-08-18

**Authors:** Jianming Sun, Linlin Li, Xiao Pan, Yini Yao, Tiantian Wang, Hui Liu, Lei Liu, Zhao Li, Xiaofang Wang

**Affiliations:** 1 Department of Packaging Engineering, Henan University of Science and Technology, Luoyang, China; 2 Department of Light Chemical Engineering, Henan Vocational College of Light Industry, Zhengzhou, China; 3 Henan Intelligent and Protective Packaging Design Engineering Research Center, Henan Luoyang, China; 4 Henan Inspection and Testing Institute Group Co., Ltd., Zhengzhou, China; National Institute of Food Technology Entrepreneurship and Management, INDIA

## Abstract

Tomatoes with rich in nutrients are very popular for humans, but they are extremely perishable during storage and transportation, which may limit their economic and nutritional value. In this research, the suitable concentration of essential oil and soaking time were investigated and selected by measuring the changes of tomatoes’ weight loss ratio and hardness with single factor experiment. Subsequently, the Box-Behnken experimental design model from Response Surface Methodology (RSM) was employed to optimize key process parameters for tomato preservation, with three independent variables selected: cinnamon essential oil concentration, konjac glucomannan (KGM) concentration, and immersion time.

As a result, cinnamon essential oil with good preservation effect was selected and combined with KGM to prepare coating materials for the preservation of tomatoes. The optimized preservation condition for tomatoes was in a solution with 0.70 g/L cinnamon essential oil, 8.20 g/L KGM and soaking time for 3.2 min, which had a good preservation effect on tomatoes with 2.5% weight loss ratio and 1.3% hardness after 10 days at room temperature, reduced the consumption of total soluble solid (TSS), delayed the decline of vitamin C (Vc) content, inhibited the increase of malondialdehyde (MDA) content, and enhanced peroxidase (POD) activity. This study demonstrates that the composite coating combines synergistic barrier properties, dual functionality, and economic advantages, establishing a theoretical framework for sustainable tomato preservation while providing transferable strategies for other produce.

## 1. Introduction

Tomatoes are welcome among consumers owing to their rich nutrients, which has been widely used in raw, cooked or processed products [1,2]. Unfortunately, tomatoes are highly susceptible to spoilage and deterioration during storage and transportation, which threatens human health. In addition, with the increasing concern about the environmental protection and food safety, the adoption of certain preservation techniques is essential to extend the freshness of tomatoes [3–5]. Consequently, it is necessary to search for an alternative packaging with the advantages of greenness, safety, non-pollution [6,7].

In recent years, many works have concentrated on extending the shelf life of tomatoes with biodegradable and edible materials such as plant essential oils and edible films. Because of the good film-forming property of Konjac glucomannan (KGM) and good biological activity of plant essential oils, plant essential oil combined with KGM in fruits and vegetables preservation has attracted the attention of many scholars.

As a water-soluble polysaccharide extracted from konjac tubers, KGM is purified as a white substance with powder, without special taste, easy to degrade and has a gel, film-forming, water absorption, thickening and other physical and chemical properties [8]. After being coated by KGM, the external surface of tomatoes can form a transparent film, which can reduce the ratio of CO_2_ release from the inside of the tomatoes due to respiration, thus preventing the external O_2_ from contacting with the inside of the fruits [9]. This reduces the respiration and transpiration of tomatoes, and inhibits the loss of nutrients and water [10,11]. In addition, it can improve the sensory and nutritional quality of tomatoes by reducing the invasion of exogenous pathogenic bacteria, and prolong the storage and preservation period of tomatoes [12–14]. Pure KGM film possesses good water vapor barrier properties, but with relatively weak antioxidant and antibacterial properties.

Plant essential oils, a kind of oil-like viscous liquid which is extracted from the roots, stems, leaves and other parts of the plant and often used for retaining food freshness, have broad-spectrum antibacterial and antioxidant properties, can inhibit the synthesis of bacterial nucleic acids by directly damaging the cell walls and cell membranes of bacteria [15–18], and can block the free radicals and inhibit lipid peroxidation [19–23].

In this study, the effects of plant essential oil on the quality of tomatoes were studied firstly, and the concentrations of plant essential oil with good preservation effects on tomatoes were selected by using weight loss ratio and hardness as evaluation indexes. Then, the concentration of essential oil, KGM concentration and soaking time with good preservation effects were determined by using the single-factor test method in advance, and the Box-Behnken RSM was used to optimize the composite coating film to obtain the best [10,24,25]. The RSM of composite coating preservation was established to optimize the composite coating agent by using weight loss ratio and hardness as indicators. Finally, the effect of KGM composite coating preservation agent obtained by response surface optimization on the nutritional quality and physiological and biochemical indexes of tomatoes during storage at room temperature was investigated, and the changes in weight loss ratio, hardness, TSS content, Vc content, MDA content and POD activity of tomatoes and the mechanisms of their effects were further investigated [11,26].

Compared to single-component coatings, the composite coating developed in this study significantly enhances preservation efficacy through three key advantages: Formation of a denser film that achieves a synergistic barrier effect; Dual functional extension, combining antimicrobial and antioxidant properties; Optimized technical cost-effectiveness. The use of green and safe composite materials in this study provides a certain research basis for the development of fruits and vegetables fresh-keeping packaging. To some extent, it reduces the global white pollution caused by plastic packaging and alleviates the global environmental pressure.

## 2. Materials and methods

### 2.1 Materials

The cherry tomato cultivar ‘Qianxi’ (Solanum lycopersicum var. cerasiforme) used in this study was sourced from Mengjin County, Henan Province, China. During the trial, the fruits were at their mature stage (the optimal phase for fresh consumption) with firm texture. Samples were selected based on morphological similarity, uniform size, comparable maturity, and absence of surface mechanical damage caused by external forces.[27]. Cinnamon essential oil, clove essential oil and mugwort essential oil (Single Essential Oil Purity ≥ 99%) were provided from Kangchun Spice Co., Ltd, Hubei, China. Konjac glucomannan was purchased from Johnson Konjac Co., Ltd, Hubei, China. Sodium hydroxide, oxalic acid, anhydrous sodium acetate, glacial acetic acid, thiobarbituric acid (TBA), trichloroacetic acid (TCA), polyvinyl pyrrolidone (PVPP), polyethylene glycol 6000(PEG 6000), triton x-100 (Triton X-100), guaiacol and 30% hydrogen peroxide, glycerol and Tween-80 were provided from De’en Chemical Reagent Co., Ltd, Tianjin, China. All the chemicals employed here were reagent grade.

### 2.2 Apparatus

The electronic balance (HZK-JA320) was provided from Huazhi Scientific Instruments Co., Ltd, Fujian, China. GY-4 fruit hardness tester was purchased from Topunnong Technology Co., Ltd, Zhejiang, China. Digital refractometer (LH-B55) was provided from Luheng Biotechnology Co., Ltd, Hangzhou, China. TDS meters (AR8011) were purchased from Sigma Instruments Co., Ltd. UV-Visible Spectrophotometer (UV759CRT) was provided from Youke Instrument Co., Ltd, Shanghai, China. High-speed frozen centrifuge (TGL-16) was purchased from Shuke Instrument Co., Ltd, Sichuan, China. Constant temperature and humidity chamber (HD-100) was provided from Dongguan Haida Instruments Co., Ltd, Zhejiang, China. Constant temperature heating magnetic stirrer (DF-101S) was purchased from Gongyi Yuhua Instrument Co., Ltd, Henan, China. Handheld Pipettes was provided from Eppendorf AG, Germany.

### 2.3 Screening of plant essential oils

Appropriate amounts of essential oils (cinnamon, clove, and mugwort) were mixed with 2 g Tween-80, then dissolved in 1 L distilled water to prepare solutions with concentrations of 0.25 g/L, 0.50 g/L, 1.00 g/L, and 2.00 g/L, respectively. The untreated group served as Control 1 (labeled CK1), while 2 g/L Tween-80 solution was designated as Control 2 (labeled CK2). The methods for determining weight loss and firmness were as follows.

(1) Weight loss ratio

The weight loss ratio was obtained by weighing the tomatoes before experiment and after storage (the experiment was repeated three times) according to equation (1).


$$Weight\;loss\;ratio(\%)=\frac{m_{0}-m_{1}}{m_{0}}\times 100\%$$ (1)

Where *m*_0_ represents the weight before weight loss, and *m*_1_ represents the weight after weight loss.

(2) Hardness

Hardness was measured with a GY-4 fruit hardness tester. During testing, at least 1 cm^2^ of the peel was peeled at three equally spaced locations in the largest part of the tomato diameter in turn, and a hardness tester with a probe size of φ11 mm was selected for testing, and the probe was always perpendicular to the peeled area and slowly and uniformly pressed to the 10 mm scale, and the hardness data were recorded in N/cm². Tests needed to be carried out for three times.

### 2.4 The preparation of KGM composite film

**Fig 1:**
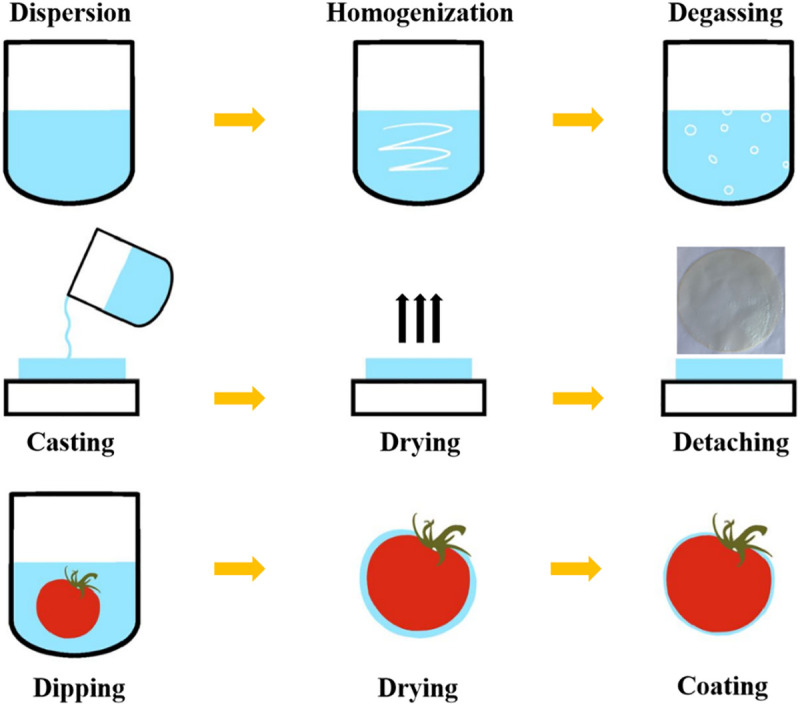
The process of KGM composite film preparation.

Fig 1 shows the process of KGM composite film preparation. A certain amount of KGM was dissolved in distilled water and stirred at 50°C and 300 r/min for 1.5 h by using constant temperature heating magnetic stirrer to form KGM film liquid. Then a certain amount of plant essential oil was added to the film liquid and emulsified with Tweene-80. The mixture was stirred at room temperature for 0.5 h and defoamed for 2 h. After natural drying, KGM composite film can be formed.

#### 2.4.1 Single factor experiment.

Single factor experiments were conducted with cinnamon essential oil (CEO) concentration, KGM concentration, and immersion time as independent variables. The effects of parameter levels on tomato weight loss and firmness were analyzed to determine the optimal parameter ranges. See Table 1 for the specific membrane liquid composition and mass fraction.

**Table 1:** Composition and quality fraction of membrane fluid.

Factor	1	2	3	4
Cinnamon essential oil concentration/(g/L)	0.25	0.5	1.0	2.0
KGM concentration/(g/L)	3	6	9	12
Soaking time/(min)	1	2	3	4

#### 2.4.2 Establishment of the RSM model for weight loss ratio and hardness.

RSM was used as an experimental design model to optimize the key process parameters for the investigation of tomatoes preservation. Three independent process variables, i.e., Cinnamon essential oil concentration (A), KGM concentration (B) and Soaking time (C), were chosen to investigate the weight loss ratio and hardness of tomatoes. This model was coded a three-level factorial (−1, 0, and 1), which was superimposed by the center points (coded 0). The range of design factor was set according to the range of Cinnamon essential oil concentration, KGM concentration and soaking time for tomatoes (Table 2).

**Table 2:** Design factor range and levels (coded).

Factors	Range and levels (coded)	
		-1	0
1	Cinnamon essential oil concentration/(g/L)	0.25	0.50
1.00	KGM concentration/(g/L)	6.00	9.00
12.00	Soaking time/(min)	2.0	3.00	4.00

### 2.5 Characterization of KGM composite film properties

#### 2.5.1 Mechanical properties measurement.

The tensile strength (TS) and elongation at break (EB) were measured using a universal testing machine. Samples were cut into 100 mm × 20 mm strips and tested at a crosshead speed of 50 mm/min, with six replicates per group. The formulas are as follows:


$$TS=\frac{F}{dh}$$ (1)

where: TS: Tensile strength (MPa); F: Maximum load at break (N); d: Film thickness (mm); h: Film width (mm).

$EB=\frac{L_{m}-L_{0}}{L_{0}}\times 100\%$ (2)

where: EB: Elongation at break (%); Lₘ: Final gauge length at break (mm); L₀: Initial gauge length (mm).

#### 2.5.2 Thickness measurement.

Film thickness was measured using a digital micrometer. Six random points were selected for each sample. The film was placed on the lower anvil, and the presser foot was gently lowered until contact was made. Readings were recorded in mm.

#### 2.5.3 Water vapor permeability (WVP).

WVP was determined by the gravimetric method. An 80-mm film disc was sealed on a permeation cup containing 10 g silica gel, then placed in a climatic chamber (38°C, 90% RH) for 72 h. Weight gain was recorded every 24 h (triplicate tests). The formula is:


$$WVT=\frac{\Delta G}{\Delta ts}$$ (3)

where: WVP: Water vapor permeability (g·h ⁻ ¹·m ⁻ ²); ΔG: Weight gain (g); Δt: Time interval (h); S: Film area (m²).

#### 2.5.4 Water solubility.

Film samples (20 mm × 20 mm) were dried at 50°C for 24 h, cooled to room temperature (RT), and weighed (m₁). They were then immersed in 50 mL distilled water (23°C, 24 h), redried (50°C, 24 h), and reweighed (m₂). Water solubility (W₂) was calculated as:


$$Ws=(m_{1}-m_{2})/m_{2}\times 100\;\%$$ (4)

where: m_1_: Mass of the dried film before immersion (g); m_2_: Mass of the dried film after water absorption and redrying (g).

#### 2.5.5 Antibacterial activity.

Escherichia coli was used as the test strain. A 0.1 mL bacterial suspension was spread on solid agar, and film samples were placed on the surface. The diameter of inhibition zones was measured using the cross method after 24 h incubation at 37°C.

#### 2.5.6 Antioxidant activity (DPPH assay).

Films (100 mg) were dissolved in 10 mL ethanol (dark, 2 h). Then, 1 mL extract was mixed with 1 mL DPPH solution (0.2 mmol/L), incubated in the dark (30 min), and absorbance was measured at 517 nm (ethanol as blank). Radical scavenging activity (%) was calculated as:


$$DPPH=\frac{A_{1}-A_{0}}{A_{1}}\times 100\;\%$$ (5)

where: A_1_: Absorbance of the control sample; A_0_: Absorbance of the test sample.

#### 2.5.7 Scanning electron microscopy (SEM).

Surface and cross-sectional morphologies of KGM films were observed using SEM. Samples (10 mm × 10 mm) were mounted on conductive tape, sputter-coated with gold, and imaged at 5 KV.

#### 2.5.8 Fourier transform infrared spectroscopy (FTIR).

FTIR spectra were recorded in ATR mode (400–4000 cm ⁻ ¹, 32 scans, 4 cm ⁻ ¹ resolution).

### 2.6 Characterization of the preservative effects of KGM composite films on tomatoes

#### 2.6.1 TSS content.

The appropriate amount of tomatoes pulp was taken, ground into juice and filtered. The filtered juice was taken in drops into a digital refractometer and tested for readings. The experiment needed to repeat three times.

#### 2.6.2 Vc content.

**Fig 2:**
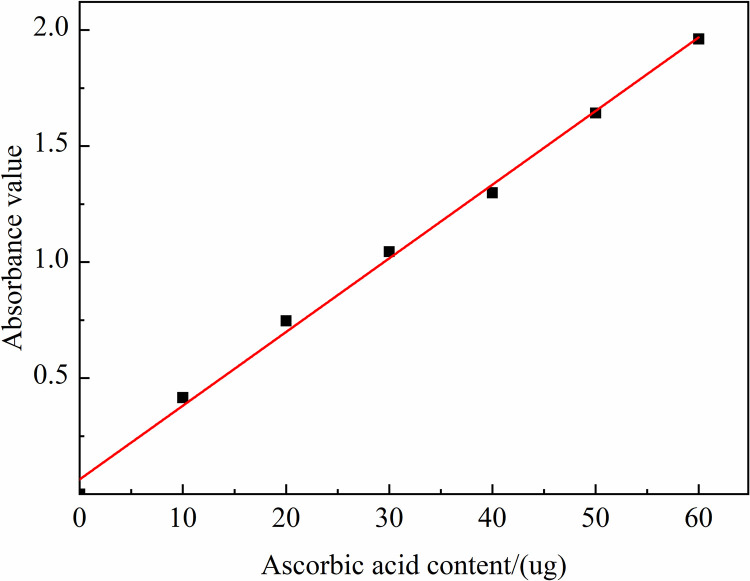
Ascorbic acid standard curve.

Firstly, the maximum absorption wavelength λ of ascorbic acid was obtained by experiment at 242 nm, and the standard curve of ascorbic acid content and absorbance value was measured and plotted at this wavelength, as shown in Fig 2.

Then, 10 g of tomatoes peeled pulp was added into the 10 ml of 5 g/L oxalic acid solution, ground using mortar and pestle. The mixture was diluted to 100 mL with distilled water, filtered at rest and the filtration was collected. 2.0 mL of the sample was extracted in a quartz cuvette and the absorbance value at 242 nm was recorded using oxalic acid solution as reference. The experiment needed to repeat three times. Based on the absorbance values obtained from the assay, the corresponding ascorbic acid content in the mixture could be found on the standard curve, and then the Vc content in tomatoes tissue could be calculated according to equation (2), and the results are expressed in mg/100 g (sample) as follows [28,29].

$V_{c}(mg/100g)=\frac{V\times m}{V_{s}\times W\times 1000}\times 100$ (2)

Where *Vc* represents the content of Vitamin C, *m* represents the quality of ascorbic acid, *V* represents total volume of sample extracts, *V*s represents Volume of sample extraction solution used for timing, *W* represents Sample Quality.

#### 2.6.3 POD activity.

POD activity was measured as follows [30]. 4.0 g of peeled tomatoes pulp was weighed with 4.0 mL of extraction buffer (containing l mmol PEG, 40 g/L PVPP and 10 g/L Triton X-100) in a mortar and grind well, then centrifuged at 4°C for 15 min at 12000 × g in a high-speed refrigerated centrifuge, and the supernatant was collected. 2.0 mL of 25 mmol/L guaiacol solution, 0.1 mL of enzyme extract and 0.1 mL of 0.5 mol/L H_2_O_2_ solution were added respectively to the cuvette, rapidly mixed before timing, the absorbance value of the extract was recorded at 470 nm every 30 s. The experiment was repeated three times with distilled water as the reference solution. The curve of the change in absorbance value of OD_470_ with time was plotted to determine the part of the curve that started to show linearity, and the value of the change in absorbance per unit time was calculated. ∆OD_470_ was calculated as shown in equation (3).

$\Delta OD_{470}=\frac{OD_{470F}-OD_{470I}}{t_{F}-t_{I}}$ (3)

Where *OD*_470F_ represents absorbance termination value of the reaction mixture, *OD*_470I_ represents initial value of absorbance of the reaction mixture, *t*_F_ represents reaction termination time, *t*_I_ represents reaction initial time.

#### 2.6.4 MDA content.

2.0 g of peeled tomatoes pulp was added to 4.0 mL of 100 g/L TCA solution in a mortar and grind well, then centrifuged for 15 min at 4°C at 10000 × g in a high-speed refrigerated centrifuge, and the supernatant was collected. The supernatant was added with 1.0 mL of 6.7 g/L TBA solution and 1.0 mL of 100 g/L TCA solution as the control, and then mixed thoroughly and left in boiling water for 15 min, then removed and cooled rapidly, transferred to a cuvette and measured the absorbance values at 450 nm, 532 nm and 600 nm, respectively. The test was repeated three times. The MDA content per gram of tomatoes (fresh weight FW) was then calculated according to equation (4), presented in mmol/g FW.

$\begin{array}{c} MDA(mmol/gFW)=\frac{[6.45\times (OD_{450}-OD_{532})-0.56\times OD_{450}]\times V}{V_{s}\times m} \end{array}$ (4)

Where MDA represents malondialdehyde content, *V* represents total volume of sample extracts, *V*_s_ represents volume of sample extracts taken at the time of measurement, *m* represents sample Quality.

### 2.7 Statistical analysis

All samples were prepared and tested at least in triplicate. Statistical analysis was conducted using Origin 2018 (origin Lab corporation, Northampton MA). Analysis of variance was performed using ANOVA procedures of the IBM SPSS software (version 26.0, IBM Inc., USA, 2012) by LSD at significance level *P* < 0.05. Tukey’s post-hoc test was used for one-way ANOVA to compare the differences between groups.

## 3 Results and discussion

### 3.1 Plant essential oil screening results

#### 3.1.1 Effect of different concentrations of essential oils on weight loss ratio of tomatoes.

The data on the weight loss ratio of tomatoes treated with different types and concentrations of essential oils were shown in Table 3. Harvested tomatoes continue to undergo respiration, consuming their own moisture and nutrients, leading to an increasing trend in weight loss rate during storage [31]. Starting from day 6, significant differences (**P <* *0.05) were observed among the different concentrations of all three essential oils. The experimental results showed that among the different concentrations of cinnamon oil, 0.50 g/L cinnamon oil effectively reduced the tomatoes weight loss ratio [32–34]. The 0.50 g/L clove oil treatment group consistently exhibited the lowest weight loss ratio throughout storage compared to other groups. Compared to cinnamon oil and clove oil, the mugwort essential oil groups generally had higher weight loss ratio overall [35–37]; however, 1.00 g/L mugwort essential oil effectively reduced tomato weight loss ratio [38,39].

**Table 3:** Effect of different essential oils on weight loss ratio of tomatoes.

		Time/ d				
		2	4	6	8	10
Cinnamon essential oil	0.25g/L	0.179 ± 0.02	0.381 ± 0.08	0.821 ± 0.04ab	1.080 ± 0.02a	1.405 ± 0.04b
	0.50g/L	0.163 ± 0.09	0.363 ± 0.10	0.733 ± 0.02c	0.953 ± 0.05B	1.345 ± 0.03c
	1.00g/L	0.169 ± 0.05	0.432 ± 0.04	0.768 ± 0.03bc	1.035 ± 0.04ab	1.399 ± 0.02b
	2.00g/L	0.175 ± 0.08	0.464 ± 0.02	0.877 ± 0.07a	1.073 ± 0.06a	1.463 ± 0.02a
Clove essential oil	0.25g/L	0.169 ± 0.03bc	0.399 ± 0.05ab	0.884 ± 0.00b	1.101 ± 0.09bc	1.460 ± 0.03ab
	0.50g/L	0.152 ± 0.02c	0.302 ± 0.04c	0.835 ± 0.04b	1.039 ± 0.05c	1.396 ± 0.03B
	1.00g/L	0.192 ± 0.02ab	0.346 ± 0.05bc	0.932 ± 0.03b	1.223 ± 0.08b	1.452 ± 0.03a
	2.00g/L	0.212 ± 0.00a	0.426 ± 0.03a	1.075 ± 0.11a	1.386 ± 0.07a	1.501 ± 0.04a
Mugwort essential oil	0.25g/L	0.128 ± 0.01ab	0.424 ± 0.01a	1.141 ± 0.09a	1.204 ± 0.05a	1.568 ± 0.06a
	0.50g/L	0.148 ± 0.01a	0.412 ± 0.02a	0.910 ± 0.08Bc	1.150 ± 0.03a	1.491 ± 0.04ab
	1.00g/L	0.109 ± 0.01B	0.339 ± 0.05B	0.814 ± 0.10C	1.025 ± 0.06B	1.441 ± 0.02B
	2.00g/L	0.124 ± 0.02ab	0.424 ± 0.03a	1.084 ± 0.11ab	1.115 ± 0.10ab	1.535 ± 0.03a

Note: The data in the table are the means ± SD of three replicates, and different lowercase letters in the same column indicate significant differences between the two data (*P* < 0.05), and different capital letters in the same column indicate highly significant differences between the two data (*P* < 0.01).

#### 3.1.2 Effect of different concentrations of essential oils on the hardness of tomatoes.

The data on tomatoes hardness after treatment with different types and concentrations of essential oils were shown in Table 4. Tomatoes hardness showed a decreasing trend during storage. This is because after harvest, the protopectin in tomatoes is broken down, causing the cell wall structure to loosen and lose viscosity, resulting in surface shrinkage and softening of the fruit tissue. Starting from storage day 4, significant differences (**P <* *0.05) were observed among the different concentrations of cinnamon oil groups, and the same was true for the different concentrations of clove oil groups. Among them, the 0.50 g/L cinnamon oil treatment group maintained a relatively high level of hardness throughout the entire storage period. The hardness of the 0.50 g/L clove oil group was consistently higher than that of other concentration treatment groups. Significant differences (**P <* *0.05) in hardness values emerged among the mugwort essential oil treatment groups starting from day 6, and the 1.00 g/L mugwort essential oil treatment group had higher hardness values [40] and not significantly different from the 0.50 g/L and 2.00 g/L treatment groups (*P* < 0.05) [41–43].

**Table 4:** Effect of different essential oils on tomatoes hardness.

		Time/ d				
		2	4	6	8	10
Cinnamon essential oil	0.25g/L	28.200 ± 0.69	26.667 ± 0.61b	23.067 ± 0.31b	21.000 ± 0.20b	16.667 ± 0.42a
	0.50g/L	28.600 ± 0.53	27.466 ± 0.12a	24.200 ± 0.20a	21.467 ± 0.12a	17.267 ± 0.31a
	1.00g/L	28.467 ± 0.42	26.933 ± 0.31ab	23.733 ± 0.31ab	21.067 ± 0.12ab	16.733 ± 0.31a
	2.00g/L	28.200 ± 0.72	26.400 ± 0.20b	23.200 ± 0.60b	20.400 ± 0.40C	16.067 ± 0.23B
Clove essential oil	0.25g/L	28.067 ± 1.21	26.067 ± 0.46b	22.133 ± 0.46Bc	19.733 ± 0.31Bc	16.067 ± 0.46bc
	0.50g/L	28.800 ± 2.27	26.933 ± 0.31a	23.200 ± 0.40a	20.733 ± 0.42a	16.800 ± 0.20a
	1.00g/L	28.467 ± 0.81	26.333 ± 0.12ab	22.667 ± 0.12ab	20.267 ± 0.12ab	16.400 ± 0.00ab
	2.00g/L	28.200 ± 0.4	25.733 ± 0.31b	21.800 ± 0.20c	19.600 ± 0.35c	15.600 ± 0.35c
Mugwort essential oil	0.25g/L	27.200 ± 0.53	25.000 ± 0.20	21.600 ± 0.20b	18.867 ± 0.31b	15.000 ± 0.20B
	0.50g/L	27.800 ± 0.60	25.533 ± 0.42	21.867 ± 0.12b	19.200 ± 0.20ab	15.400 ± 0.53ab
	1.00g/L	28.200 ± 0.20	26.000 ± 0.53	22.467 ± 0.42a	19.600 ± 0.35a	16.133 ± 0.50a
	2.00g/L	27.467 ± 0.12	25.264 ± 0.50	21.733 ± 0.31b	19.067 ± 0.12b	15.533 ± 0.23ab

Note: The data are the same as Table 3.

#### 3.1.3 Effect of three optimal essential oil concentrations on weight loss of tomatoes.

The control groups CK1 (the untreated group as control group 1) and CK2 (2 g/L of Tween-80 as control group 2) were introduced, the data analysis of the best treatment groups of cinnamon, clove and mugwort essential oils on the weight loss ratio of tomatoes were shown in Table 5. Compared with the control group, each best essential oil treatment group suppressed the increase of weight loss ratio, in which the weight loss ratio of 0.50 g/L cinnamon essential oil treatment group was at a lower level overall during the storage period and the change was relatively stable. From day 6 onwards, the overall difference between the groups was significant (*P* < 0.05). On day 10, the lowest weight loss ratio was observed in the 0.50 g/L cinnamon essential oil treatment group, which was highly significant (*P* < 0.01) from CK1, CK2 and 1.00 g/L mugwort essential oil treatment groups. Compared to CK1 and CK2, the weight loss ratio of cinnamon essential oil group was reduced by 10.87% and 9.85%, respectively. Therefore, the 0.50 g/L cinnamon essential oil was the most suitable concentration of essential oil in terms of the magnitude of the weight loss ratio values and the cost of materials [44,45].

**Table 5:** Effect of three optimal essential oil concentrations on weight loss ratio of tomatoes.

	Time/ d				
	2	4	6	8	10
CK1	0.217 ± 0.08	0.431 ± 0.16	1.075 ± 0.06a	1.219 ± 0.01a	1.509 ± 0.03a
CK2	0.156 ± 0.05	0.481 ± 0.02	1.054 ± 0.07a	1.256 ± 0.05a	1.492 ± 0.03ab
0.50g/L Cinnamon essential oil	0.163 ± 0.09	0.363 ± 0.10	0.733 ± 0.02c	0.953 ± 0.05B	1.345 ± 0.03D
0.50g/L Clove essential oil	0.152 ± 0.02	0.302 ± 0.04	0.835 ± 0.04b	1.039 ± 0.05c	1.396 ± 0.03 Cd
1.00g/L Mugwort essential oil	0.109 ± 0.01	0.339 ± 0.05	0.814 ± 0.10C	1.025 ± 0.06B	1.441 ± 0.02bc

Note: The data are the same as Table 3.

#### 3.1.4 Effect of three optimal essential oil concentrations on tomatoes hardness.

The results of data analysis on tomatoes hardness between the best treatment groups of cinnamon, clove and mugwort essential oils and the two CK groups are shown in Table 6. Compared with the control group, each best essential oil treatment group effectively maintained the hardness of tomatoes, with the overall hardness of the 0.50 g/L cinnamon essential oil treatment group at a higher level and relatively stable changes. From day 4, the overall difference between the groups was significant (*P* < 0.05). On day 10, the hardness was highest in the 0.50 g/L cinnamon essential oil treatment group and was highly significant (*P* < 0.01) from all other fractions except for no significant difference with the 0.50 g/L clove essential oil group (*P* > 0.05), which increased by 8.37% and 10.69% compared to CK1 and CK2, respectively. Therefore, 0.50 g/L of cinnamon essential oil was more effective in maintaining the hardness of tomatoes.

**Table 6:** Comparative effects of three optimal essential oil concentrations on tomatoes hardness.

	Time/ d				
	2	4	6	8	10
CK1	27.267 ± 0.12	25.200 ± 0.50c	21.800 ± 0.31d	19.133 ± 0.12d	15.933 ± 0.50c
CK2	27.000 ± 0.31	25.000 ± 0.40c	21.533 ± 0.20d	19.467 ± 0.60 Cd	15.600 ± 0.20c
0.50g/L Cinnamon essential oil	28.600 ± 0.53	27.466 ± 0.12a	24.200 ± 0.20a	21.467 ± 0.12a	17.267 ± 0.31a
0.50g/L Clove essential oil	28.800 ± 2.27	26.933 ± 0.31a	23.200 ± 0.40B	20.733 ± 0.42aB	16.800 ± 0.20ab
1.00g/L Mugwort essential oil	28.200 ± 0.20	26.000 ± 0.53B	22.467 ± 0.42c	19.600 ± 0.35Bc	16.133 ± 0.50Bc

Note: The data are the same as Table 3.

In conclusion, the effect of each essential oil treatment group was more significant relative to the weight loss ratio and hardness of tomatoes in both CK groups. Compared with the different essential oil treatment groups, the difference between the cinnamon essential oil treatment group and the other two essential oil treatment groups was significant. From the quality indexes of weight loss ratio and hardness, especially 0.50 g/L of cinnamon essential oil was highly significantly different from CK1, CK2 and 1.00 g/L of mugwort essential oil (*P* < 0.01). Although it was not significantly different from 0.50 g/L of clove essential oil (*P* > 0.05), 0.50 g/L of cinnamon essential oil was more effective in reducing weight loss and effectively maintaining the hardness of tomatoes. Therefore, the final choice of 0.50 g/L cinnamon essential oil as the plant freshness preservation essential oil for tomatoes can provide the best preservation effect.

### 3.2 Preparation and optimization results of composite coating film preservation agent

#### 3.2.1 Single-factor test analysis.

**Fig 3:**
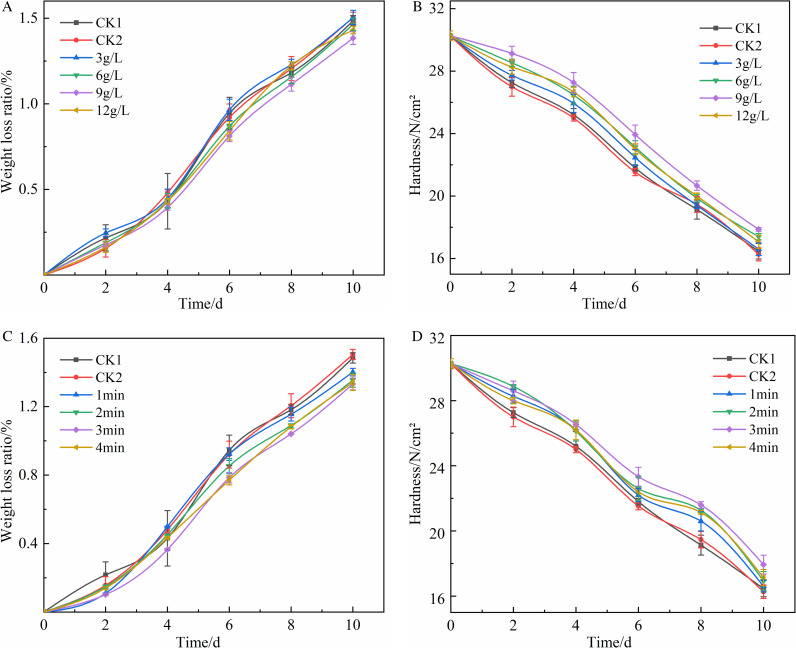
(a) The effect of KGM concentration on the weight loss ratio; (b) The effect of KGM concentration on the hardness; (c) The effect of soaking time on the weight loss ratio; (d) The effect of soaking time on the hardness.

Fig 3 represented the effect of KGM concentration and soaking time on the weight loss ratio and hardness of tomatoes during the storage period, the detailed data could be found in S1-4 Table. The weight loss ratio of each group showed an increasing trend over time, but the weight loss ratio of each experimental group was lower than that of CK1 and CK2, where the weight loss ratio of the 9.00 g/L treatment group remained at a lower level, and there was no significant difference between CK1 and CK2 (*P* > 0.05) (Fig 3a). The treatment group with KGM concentration of 9.00 g/L reduced the weight loss ratio by 14.07% and 11.74% compared with those of CK1 and CK2 groups, respectively. Among them, the treatment groups with KGM at concentration of 9.00 g/L had 6.80% and 8.04% lower weight loss ratio compared with CK1 and CK2 groups, respectively, and were highly significantly different from CK1, CK2 and 3.00 g/L groups (*P* < 0.01), significantly different from 6 g/L group (*P* < 0.05). Therefore, KGM at a concentration of 9.00 g/L could effectively reduce the weight loss ratio of tomatoes [46].

The hardness of each group showed a decreasing trend over time, but all experimental groups were higher than CK1 and CK2, with the hardness of the KGM treatment group at 9.00 g/L remaining at a higher level; there was no significant difference between CK1 and CK2 (*P* > 0.05) (Fig 3b). The KGM treatment group with a concentration of 9.00 g/L increased the hardness by 8.47% and 9.84% compared to the CK1 and CK2 groups, respectively, where there was a significant difference (P < 0.05) with each of the other fractions except for no significant difference (P > 0.05) with the 6.00 g/L treatment group. Therefore, the most suitable concentration of KGM was 9.00 g/L in terms of freshness preservation.

The weight loss ratio of each group of tomatoes showed an increasing trend with time, where the weight loss ratio of the 3.00 min soaking treatment group remained relatively low during the storage period; and there was no significant difference between CK1 and CK2 (*P* > 0.05) (Fig 3(c)). On the 10th day of storage, the weight loss ratio of the 3.00 min treatment group was reduced by 10.24% and 11.43% compared to the CK1 and CK2 groups, respectively, and was highly significantly different from the CK1, CK2 and 1.00 min groups (*P* < 0.01), and not significantly different from the 2.00 min and 4.00 min groups (*P* > 0.05). Therefore, the treatment group with 3.00 min soaking time could effectively reduce the weight loss ratio of tomatoes.

The hardness of each group showed a decreasing trend with time, with the hardness of the 3.00 min soaking treatment group remaining at a higher level after the 4th day of storage. And there was no significant difference between CK1 and CK2 (*P* > 0.05) (Fig 3(d)). On the 10^th^ day of storage, the hardness of the 3.00 min soaking treatment group increased by 8.90% and 10.24% compared to the CK1 and CK2 groups, respectively, and were highly significantly different from CK1 and CK2 (*P* < 0.01). Therefore, the soaking time of 3.00 min was determined to be the best time in terms of time cost, weight loss ratio and preservation effect.

#### 3.2.2 Response surface test optimization and results.

In order to investigate the effect of the interaction of three factors on the freshness of tomatoes and predict the optimal ratios of the three factors, a single-factor test method was used to select the three levels with a greater effect on weight loss ratio and hardness respectively based on the Box-Behnken design module in the software Design Expert 12. The tests were coded −1, 0 and 1 for the 3 levels respectively and the design combinations were shown in Table 7.

**Table 7:** Box-Behnken experimental design portfolio.

Run	A(g/L)	B(g/L)	C(min)	Response Value	
				Y1(%)	Y_2_(N/cm^2^)
1	1	0	−1	1.31	16.93
2	1	1	0	1.42	15.47
3	0	1	−1	1.47	15.20
4	0	0	0	1.17	17.87
5	0	−1	1	1.20	16.80
6	−1	−1	0	1.39	15.60
7	0	0	0	1.09	18.00
8	0	0	0	1.14	18.20
9	−1	0	−1	1.37	15.93
10	0	0	0	1.16	18.53
11	0	0	0	1.07	18.13
12	−1	1	0	1.50	15.00
13	0	1	1	1.41	15.67
14	1	0	1	1.23	17.20
15	1	−1	0	1.19	16.53
16	−1	0	1	1.37	16.20
17	0	−1	−1	1.27	16.60

Since the effects of A (cinnamon essential oil concentration), B (KGM concentration), and C (soaking time) on weight loss ratio (Y_1_) and hardness (Y_2_) of tomatoes were non-linear, in order to determine the effect of each factor on the magnitude of the response values, multiple quadratic regressions were fitted to the experimental results of weight loss ratio and hardness to establish the respective binary regression model equations as shown in equations (5) and (6) [47].

Y1 = 1.09−0.0586A + 0.0985B-0.0281C + 0.0265AB-0.0153 AC + 0.0015BC + 0.1539A^2^+0.1332B^2^+0.0765C^2^[5]

Y2=18.43+0.425A-0.5376B+0.1492C-0.0761AB+0.0052AC+0.0668BC-1.28A^2^-1.5B^2^-0.5817C^2^[6]

#### 3.2.3 Response surface analysis of each factor on weight loss ratio.

The regression model equations for weight loss ratio were subjected to ANOVA and plausibility analysis, and the results are shown in Table 8 and 9 [48–50].

**Table 8:** Variance analysis results of regression model for weight loss ratio.

Source	Sum of squares	Degree of freedom	Mean Square	F	*P*
Models	0.2820	9	0.0313	23.17	0.0002**
A	0.0275	1	0.0275	20.33	0.0028**
B	0.0738	1	0.0738	54.57	0.0002**
C	0.0060	1	0.0060	4.42	0.0736
AB	0.0030	1	0.0030	2.19	0.1824
AC	0.0010	1	0.0010	0.7323	0.4204
BC	9.000E-06	1	9.000E-06	0.0067	0.9373
A^2^	0.0744	1	0.0744	55.02	0.0001**
B^2^	0.0747	1	0.0747	55.26	0.0001**
C^2^	0.0246	1	0.0246	18.21	0.0037**
Residual	0.0095	7	0.0014		
Misfitting term	0.0024	3	0.0008	0.4640	0.7229
Pure error	0.0070	4	0.0018		

Note: *P* < 0.01 indicates highly significant and is indicated by **; 0.01 < P < 0.05 indicates significant and is indicated by *; *P* > 0.05 indicates not significant and is not marked.

**Table 9:** Reliability analysis of the model.

mean value	standard deviation	R^2^	Adjusted R^2^	Predicted R^2^	variable coefficient C.V./%	Precision
1.28	0.036	0.9675	0.9258	0.8188	2.88	13.5127

F-test and *P*-value were used to determine statistical significance and regression coefficients. The regression model F = 23.17, *P* = 0.0002, indicating that this model was highly significant (*P* < 0.01), while the misfit term F = 0.4640, *P* = 0.7229, was not significant (*P* > 0.05), indicating that this model was reasonable within the range of independent variable factors; the correlation coefficient R^2^ = 0.9675 and the predicted R^2^ = 0.8188, the difference with the adjusted R^2^ was less than 0.2, and the predicted value was closer to the actual value, indicating that this model fitted better, the coefficient of variation was 2.88%, which was less than 5%, indicating that this model was more accurate, so the experimental data had certain reliability; the precision was 13.5127, which was better than 4, indicating that this model could be used for optimization. Therefore, this model could be used to analyze and predict the effects of various factors on weight loss ratio of tomatoes within the range of independent variable factors.

According to the magnitude of the P-values of each factor, the order of the degree of influence on the weight loss ratio of tomatoes was B > A > C. From the significance test of each coefficient, it was clear that A, B, A^2^, B^2^, and C^2^ were highly significant terms (*P* < 0.01), and the crossover terms were not significant, indicating that the effect of the interaction between the two factors was small.

**Fig 4:**
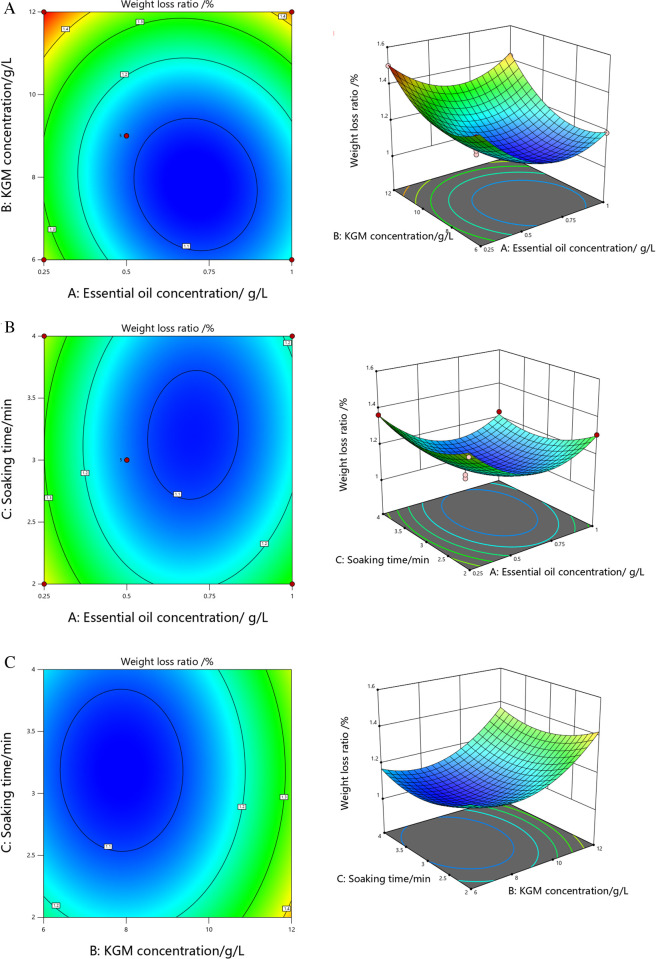
Response surface/contour plot of each interactive factor to tomatoes weight loss ratio.

Figs 4(a)-(c) showed the response surface and contour model plots of each interaction factor on weight loss ratio of tomatoes [51]. The steeper the change of the response surface was, the greater the degree of influence of the interaction of the test factors on the weight loss ratio would be. And in the contour plot of the interaction of the factors, if the contour shape is closer to a circle, it represents the weaker interaction of the factors, and the opposite is true for the ellipse, where the center of the smallest circle represents the most value of the response value. As shown in Figs 4(a)-(c), response surface and the significance of the interaction term showed that the interaction between A and B had the greatest effect on the weight loss ratio of tomatoes, followed by A and C, and the weakest was B and C. Meanwhile, contour plots showed that there was an interaction between two of the three factors, but none of them was significant, and all three factors had the lowest point in the response surface, indicating that there was a minimum in the weight loss ratio of tomatoes within the test range.

In the response surface plot of each interaction factor on the weight loss ratio of tomatoes, as shown in Fig 4(a), the weight loss ratio of tomatoes gradually showed a trend of first decreasing and then increasing with the increase of the concentration of cinnamon essential oil, because the low concentration of essential oil and the small content of its effective active ingredients could not play a big role in antibacterial and antioxidant effects; while the higher concentration of essential oil would cause some drug damage to the fruit, thus stimulating the normal metabolism inside the fruit and accelerating the aging process. As shown in Fig 4(b), the weight loss ratio of tomatoes showed a trend of decreasing and then increasing with the increase of soaking time, but the changes were not significant, indicating that the effect of soaking time on weight loss ratio was not as significant as that of cinnamon essential oil and KGM concentration. As shown in Fig 4(c), the weight loss ratio of tomatoes showed a trend of decreasing and then increasing with increasing KGM concentration. With the film formation effect was not good and the preservation effect was not significant, while with too high concentration, the film layer formed would be thicker, resulting in anaerobic respiration of tomatoes, accelerating the consumption of water and nutrients and causing weight loss.

#### 3.2.4 Response surface analysis of each factor on hardness.

The regression model equations for hardness were subjected to ANOVA and plausibility analysis, and the results were shown in Table 10 and 11.

**Table 10:** Results of variance analysis of hardness regression model.

Source	Sum of squares	Degree of freedom	Mean Square	F	*P*
Models	20.42	9	2.27	37.56	<0.0001**
A	1.45	1	1.45	23.92	0.0018**
B	2.20	1	2.20	36.36	0.0005**
C	0.1693	1	0.1693	2.80	0.1380
AB	0.0245	1	0.0245	0.4052	0.5446
AC	0.0001	1	0.0001	0.0019	0.9662
BC	0.0178	1	0.0178	0.2950	0.6039
A^2^	5.17	1	5.17	85.59	<0.0001**
B^2^	9.45	1	9.45	156.44	<0.0001**
C^2^	1.42	1	1.42	23.58	0.0018**
Residual	0.4229	7	0.0604		
Misfitting term	0.1709	3	0.0570	0.9040	0.5136
Pure error	0.2520	4	0.0630		

Note: Same as Table 8.

**Table 11:** Reliability analysis of the model.

mean value	standard deviation	R^2^	Adjusted R^2^	Predicted R^2^	variable coefficient C.V./%	Precision
16.70	0.2458	0.9797	0.9536	0.8436	1.47	17.9462

The regression model F = 37.56, *P* < 0.0001, indicating that this hardness model had a highly significant correlation (*P* < 0.01); and the misfit term F = 0.9040, *P* = 0.5136, which was not significant (*P* > 0.05); the correlation coefficient R^2^ = 0.9797, the predicted R^2^ = 0.8436, the difference with the adjusted R^2^ was less than 0.2, indicating that the predicted value was closer to the actual value, so the hardness model fitted better; the coefficient of variation was 1.47%, which was less than 5%, indicating that the hardness model was accurate and the experimental data was reliable; the precision was 17.9462, which was greater than 4, indicating that this model was good enough to be used for optimization. Therefore, this model could be used to analyze and predict the effects of various factors on tomatoes hardness within the range of independent variable factors.

**Fig 5:**
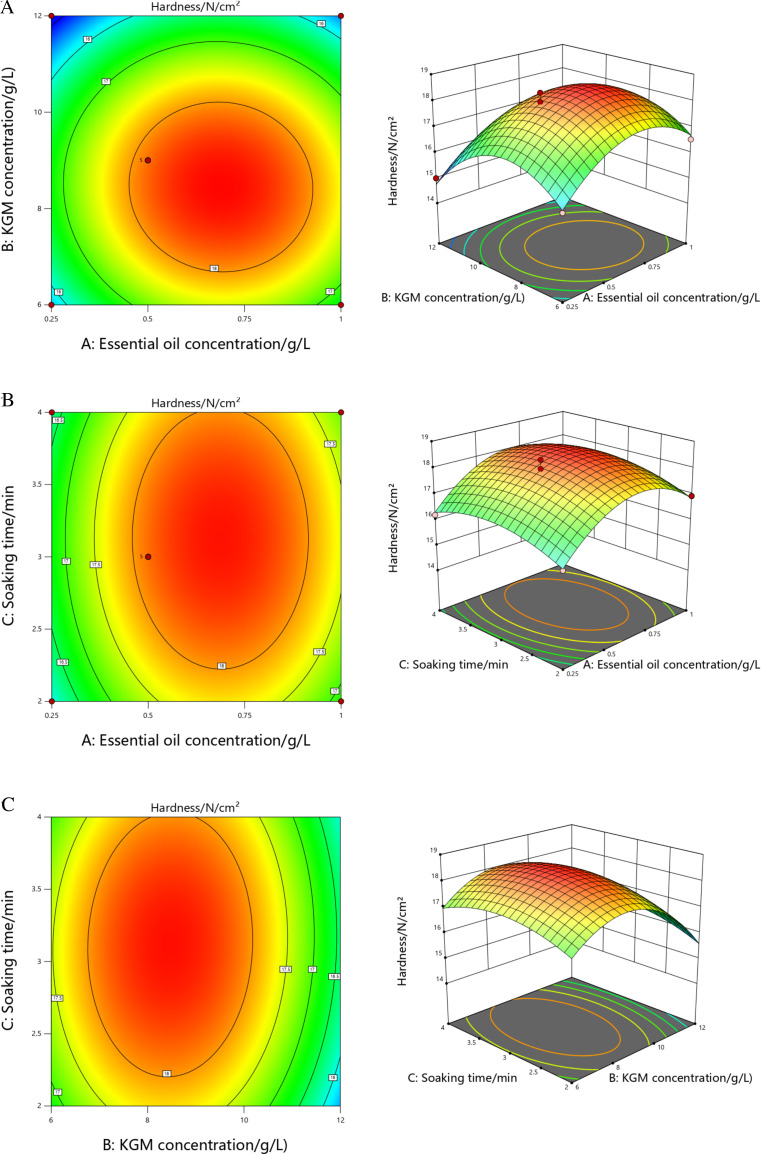
Response surface/contour plot of each interactive factor to tomatoes hardness.

According to the magnitude of the *P*-values of each factor, the order of the degree of influence of tomatoes hardness was B > A > C. From the significance tests of each coefficient, it was clear that A, B, A^2^, B^2^, and C^2^ were highly significant terms, and the crossover terms were not significant, which proved that the interaction between the two factors had little effect. Figs 5(a)-(c) showed the response surface/contour analysis plots of each factor on tomatoes hardness.

In the response surface/contour plots of each interaction factor on tomatoes hardness, as shown in the response surface of Fig 5(a)-(c), it could be seen that the interaction between the concentration of cinnamon essential oil and KGM concentration had the greatest effect on tomatoes hardness, followed by KGM concentration and soaking time, and the weakest was the concentration of cinnamon essential oil and soaking time. Also, it could be seen that there was an interaction between the three factors, but none of them was significant, and each interaction factor had the highest point in the response surface plots, indicating that there was a maximum value of hardness in tomatoes within the test range.

Among them, in the response surface plot of tomatoes hardness, as shown in Fig 5(a), the hardness of tomatoes showed a trend of first increasing and then decreasing with the increase of the concentration of cinnamon essential oil, because the ability of tomatoes to fight against the harsh environment and maintain their own physiological functions decreases during the ripening process. With low concentration of essential oil and low content of its effective active ingredients, tomatoes are easily invaded by microorganisms, which accelerates the softening and rotten deterioration of the flesh fruit; and the higher concentrations of essential oils can cause a certain amount of damage to the fruit, leading to its decay and softening. As shown in Fig 5(b), the hardness of tomatoes showed a trend of increasing and then decreasing with the increase of soaking time, but the changes were not significant, indicating that the effect of soaking time on hardness was not as significant as that of cinnamon essential oil and KGM concentration. As shown in Fig 5(c), the hardness of tomatoes showed a trend of increasing and then decreasing with the increase of KGM concentration; however, if the concentration is too high, it will lead to the formation of a thick film layer, resulting in anaerobic respiration of tomatoes and the massive accumulation of ethanol, acetic acid and other products in cell tissues, which will have a certain toxic effect on fruit cells, thus reducing fruit quality.

#### 3.2.5 Validation tests.

The regression model was solved by using the software Design-Expert 12, and the optimal results were set to obtain the minimum weight loss ratio and maximum hardness, and the optimal composite film preservative was obtained within the selected ranges of cinnamon essential oil concentration, KGM concentration, and soaking time: 0.69 g/L for cinnamon essential oil, 8.19 g/L for KGM concentration, and 3.16 min for soaking time. And the predicted weight loss ratio was 1.06% and hardness was 18.51 N/cm^2^. To facilitate the experimental operation in the actual situation, the optimal conditions were adjusted as follows: the concentration of cinnamon essential oil was 0.70 g/L, the concentration of KGM was 8.20 g/L, and the soaking time was 3.20 min, and the validation experiments were conducted, and the weight loss ratio and hardness of tomatoes were measured as 1.09% and 18.33 N/cm^2^, respectively. Under the same conditions, the relative errors were 2.50% and 1.30%, respectively, indicating that the response model could better predict the weight loss ratio and hardness of tomatoes, which verified the validity of the model.

### 3.3 Characterization and analysis of KGM composite film properties

#### 3.3.1 Analysis of mechanical properties, thickness, water vapor permeability, and water solubility of composite films.

The mechanical properties, thickness, water vapor permeability, and water solubility of the KGM composite films are presented in Table 12. Compared to the pure KGM film, the TS of the KGM composite film increased, while EB decreased. This may be attributed to hydrophobic components in cinnamon essential oil (CEO), such as cinnamaldehyde, interacting with KGM molecular chains via hydrophobic interactions and hydrogen bonding, thereby enhancing intermolecular forces and improving TS. However, these interactions may disrupt the crystalline regions of KGM, leading to reduced EB.

The thickness of the KGM composite film increased after incorporating Tween-80 and CEO, likely due to the added volume from the emulsified oil droplets.

Water vapor permeability, a key indicator of gas barrier performance, decreased in the KGM composite film. This reduction can be explained by the hydrophobic constituents of CEO replacing some KGM hydrogen bonds, thereby decreasing water adsorption sites and enhancing moisture resistance.

KGM, as a hydrophilic polymer, exhibits high water solubility. However, the water solubility of the KGM composite film decreased by 22.6% compared to the pure KGM film. This decline may result from: Hydrophobic shielding: CEO components coating KGM’s hydrophilic groups. Enhanced molecular packing: CEO molecules inserting into KGM’s amorphous regions, promoting tighter chain alignment.

#### 3.3.2 Analysis of antibacterial and antioxidant properties of composite films.

The antibacterial activity of the composite films was evaluated based on the inhibition zone diameter in agar plate assays. As shown in Table 12, the KGM composite film exhibited significantly stronger antibacterial effects against Escherichia coli (inhibition zone: 13.29 mm) compared to the pure KGM film. The limited antibacterial activity of the pure KGM film may be attributed to its hydrophilic polysaccharide structure, which lacks inherent antimicrobial functional groups. The enhanced antibacterial performance of the KGM composite film likely stems from: Sustained release of cinnamaldehyde from CEO, disrupting bacterial cell membranes. Dense film microstructure, hindering bacterial access to nutrients.

**Table 12:** Mechanical properties, thickness, water vapor permeability, and water solubility of composite and pure KGM films.

Experimental group	KGM composite film	KGM monolayer film
TS/MPa	28.63 ± 0.49	25.16 ± 0.65
EB/%	23.87 ± 0.79	30.48 ± 0.38
Thickness/mm	0.068 ± 0.005	0.052 ± 0.004
WVP/%	1.65 ± 0.04	2.31 ± 0.05
WS/%	30.73 ± 0.29	39.68 ± 0.14
IZD/mm	13.29 ± 0.59	1.75 ± 0.42
DPPH RSA/%	31.32 ± 0.89	12.46 ± 0.93

Following GB 31604.1−2015 (National Food Safety Standard: General Rules for Migration Testing of Food Contact Materials), 10% ethanol was selected as the food simulant for low-alcohol (<10%) or non-acidic foods, while 95% ethanol simulated fatty foods. The DPPH radical scavenging capacity of the films in food simulants is presented in Table 12. Key findings: The pure KGM film showed weak antioxidant activity, as its dense structure impeded radical penetration. The KGM composite film demonstrated significantly higher DPPH scavenging ability, due to: Cinnamaldehyde’s conjugated aldehyde-phenyl structure, providing active hydrogen atoms to quench radicals. Phenolic hydroxyl groups in eugenol, releasing H⁺ to reduce DPPH radicals.

#### 3.3.3 SEM analysis of KGM composite films.

**Fig 6:**
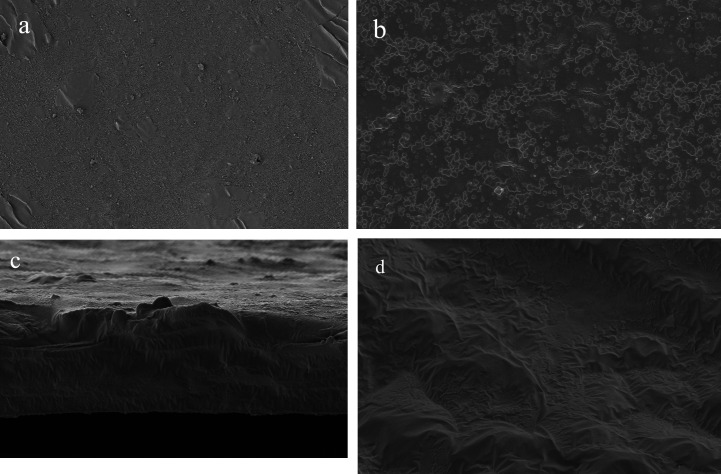
SEM of KGM composite film. Note: (**a**) and (**b**) represent the surface microstructure images of single film and composite film, respectively, while (**c**) and (**d**) represent the cross-sectional microstructure images of single film and composite film, respectively.

SEM was employed to examine the surface and cross-sectional morphology of KGM composite films and pure KGM films. As shown in Fig 6, the pure KGM film surface exhibited: Slight drying shrinkage patterns without cracks or pores Cross-section; A smooth, flat fracture surface characteristic of brittle failure. In contrast, the KGM composite film surface displayed: Uniformly dispersed essential oil droplets embedded in a continuous KGM matrix, with no phase separation cracks. Cross-section: A “hill-valley” fracture morphology with; No delamination; Dense and crack-free structure; Homogeneous molecular chain cross-linking. These observations confirm the effectiveness of Tween-80 emulsification in achieving complete essential oil incorporation within the KGM matrix.

#### 3.3.4 FTIR analysis of KGM composite films.

**Fig 7:**
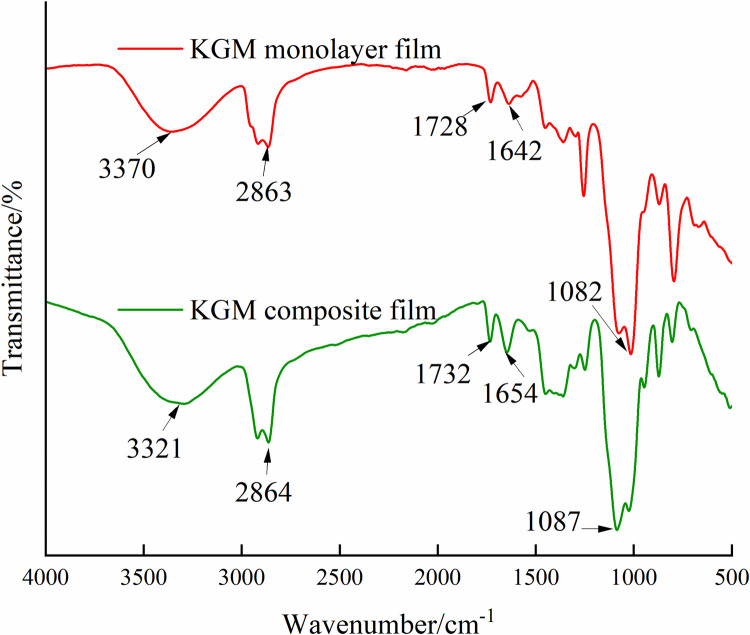
Infrared Spectra of KGM Composite Film.

The structure and interaction of each component in the KGM composite film can be judged by infrared spectroscopy. As can be seen from Fig 7, the absorption peaks at 3370 cm^-1^ and 3321 cm^-1^ are caused by the expansion vibration of -OH, and the absorption peaks at 2863 cm^-1^ and 2864 cm^-1^ are caused by the expansion vibration of -CH. The absorption peaks at 1728 cm^-1^ and 1732 cm^-1^ were caused by the C = O vibration in the acetyl group, the absorption peaks at 1642 cm^-1^ and 1654 cm^-1^ were caused by the -OH bending vibration, and the absorption peaks at 1082 cm^-1^ and 1087 cm^-1^ were caused by the C-O-C glycosidic bond vibration. Compared with the KGM single film, the peak width at about 3400 cm^-1^ decreases and the strength decreases, mainly due to the competitive hydrogen bonding of the hydrophobic components of essential oils, which causes -OH to be shielded. The intensities of the other bands increased compared with that of KGM single membrane, which were caused by the addition of -CHO, Ar-OH and -OCH_3_ components of essential oils to KGM matrix, respectively, and the overall compatibility of the three was better.

### 3.4 Results of the effect of KGM composite coating preservative on the freshness of tomatoes

**Fig 8:**
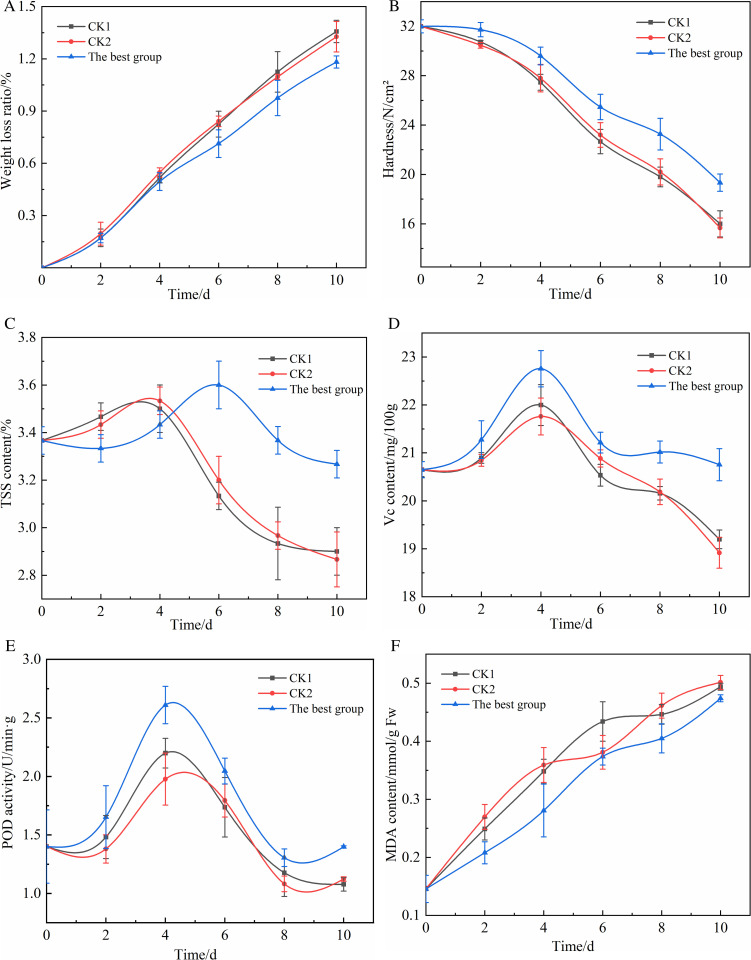
(a) Effect of KGM composite film coating preservative on weight loss ratio of tomatoes; (b) Effect of KGM composite film coating preservative on the hardness of tomatoes; (c) Effect of KGM composite film coating preservative on TSS content of tomatoes; (d) Effect of KGM composite film coating preservative on Vc content of tomatoes; (e) Effect of KGM composite film coating preservative on POD activity of tomatoes; (f) Effect of KGM composite film coating preservative on MDA content of tomatoes.

**Scheme 1:**
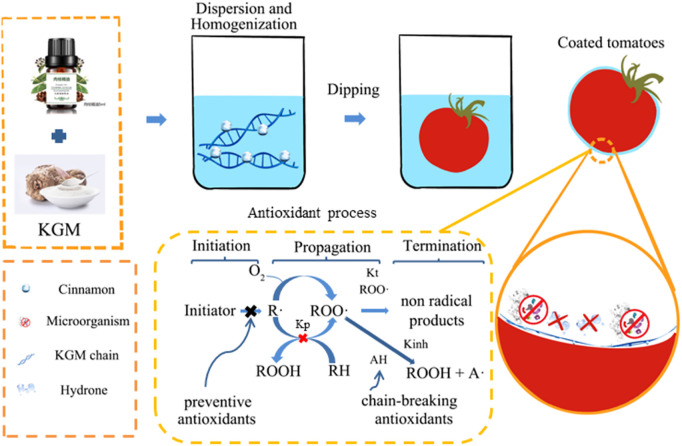
The reaction mechanism of KGM composite coating during different stages of tomatoes preservation.

The effect of KGM composite coating preservative on the freshness of tomatoes was shown in Fig 8, the detailed data can be found in Supplementary Table 5–10. Scheme 1 revealed the reaction mechanism of KGM composite coating during different stages of tomatoes preservation. It was found that transpiration and respiration were important causes of fruit weight loss ratio. After being picked, tomatoes still keep strong vital activities but lose the nutrients and water supplied by the mother and soil, and can only rely on transpiration and respiration to consume their own water and dry matter to maintain vital activities, causing natural loss of fruit, wrinkling and softening of the tomatoes skin, and eventually leading to weight loss of tomatoes [52]. Therefore, the weight loss ratio can directly reflect the freshness preservation effect of the composite coating film [53].

The effect of KGM composite coating film on the weight loss ratio of tomatoes could be seen from Fig 8(a), with the increase of storage time, the weight loss ratio of all components of tomatoes showed an increasing trend. During the whole storage period, the weight loss ratio of the optimal group was lower than that of the two control groups, and there was always no significant difference in the weight loss ratio between the two control groups, which indicating that Tween-80 had no significant effect on the weight loss ratio of tomatoes(*P* > 0.05). From day 6 onward, the weight loss ratio of the optimal group showed a more significant reduction than that of the two CK groups, probably because at the post-ripening stage, the composite coating treatment not only attenuated the material loss due to respiration and transpiration of the fruit, but the slow release of cinnamon essential oil also slowed down the decay and deterioration of tomatoes caused by the infestation of foreign pathogens, effectively reducing the weight loss ratio of tomatoes. On day 10, the optimal group showed significant differences (*P* < 0.05) from both controls, which were 13.24% and 11.28% lower compared to CK1 and CK2, respectively.

Hardness is an important index to measure the maturity and quality of the fruit during post-harvest storage, and its hardness gradually decreases during the transformation of the fruit into the mature stage or even the senescence stage.

The effect of KGM composite coating film on the hardness of tomatoes could be seen from Fig 8(b), the hardness of each component of tomatoes gradually showed a decreasing trend with the increase of storage time, and the hardness of the two control groups decreased significantly faster. During the storage period, the hardness of the optimal group was always higher than that of the two control groups. There was no significant difference between the two control groups throughout the storage period. From day 4 of storage, the hardness of the optimal group was always significantly different from that of the two CK groups (*P* < 0.05), probably because the KGM composite coating film inhibited the activity of hydrolyzed pectin substances and cellulose-related enzymes in tomatoes, making the dissolution of the gum layer and cellulose decomposition in tomatoes fruits slow and delaying the softening of tomatoes. On day 10, the hardness of CK1 and CK2 had decreased to 16.00 N/cm^2^ and 15.67 N/cm^2^, while the hardness of the optimal group was highly significantly higher than that of the CK1 and CK2 groups (*P* < 0.01) at 19.33 N/cm^2^, and the hardness of the optimal group increased by 20.81% and 23.36% compared to CK1 and CK2. Therefore, KGM composite coating film could be beneficial to maintain the hardness of tomatoes and delay the softening and rotting of flesh.

TSS content in tomatoes is an important indicator of the nutritional composition, maturity and quality status of fruits and vegetables. The TSS content of tomatoes varied as shown in Fig 8(c). With the increase of storage time, the overall TSS content of tomatoes in each component showed a trend of increasing and then decreasing [54]. During the storage period, there was no significant difference in the TSS content of the two control groups, indicating that Tween-80 had no significant effect on the TSS content of tomatoes. After day 6, the TSS content of the two CK groups decreased faster than that of the optimal group because the consumption of soluble sugars and other nutrients due to respiration of tomatoes was effectively attenuated by the treatment with the composite preservative, and the slow release of cinnamon essential oil could inhibit the growth and reproduction of pathogenic bacteria using nutrients from tomatoes. On day 10, the TSS content of the optimal group was highly significantly higher than that of the CK1 and CK2 groups (*P* < 0.01) at 3.27%, which was 12.76% and 13.94% higher in the optimal group compared to CK1 and CK2. Therefore, KGM composite coating film could effectively delay the post-ripening stage of tomatoes and had a significant inhibitory effect on the decrease of TSS content, maintaining the nutrition and flavor of tomatoes fruits.

The changes in Vc content of tomatoes could be seen from Fig 8(d), with the increase in storage time, the Vc content showed a trend of rising and then decreasing, which is due to the heat generation by the continuous respiration of tomatoes in the early period (before 4 days), resulting in a rising ambient temperature and a large amount of ethylene production, which had a ripening effect on tomatoes and promoted the rise of Vc content until the tomatoes were fully ripe, when the Vc content could reach its peak [55]. The Vc content can reach its peak when the tomatoes are fully ripe; later Vc is easily decomposed by oxidation and thus quickly loses its physiological activity and its content decreases. On day 10, the Vc content of the optimal group increased by 8.07% and 9.67%, respectively. Therefore, KGM composite coating effectively improved the antioxidant properties of tomatoes, significantly inhibited the decrease of Vc content, and maintained the nutrition and flavor of tomatoes.

Free radicals produced by cells of fruits and vegetables when aging and suffering from adversity damage can induce peroxidation of cell membrane lipids, while MDA is one of the main products of peroxidation, and the amount of its content is usually used as an indicator of peroxidation, reflecting the degree of damage and peroxidation of cell membrane lipids of fruits and vegetables [56]. As could be seen from Fig 8(e), the MDA content of each component tomatoes gradually increased with the increase of storage time, and the MDA content of the two control groups was always higher than the optimal group, which is because the active substances related to the structure of cinnamaldehyde in cinnamon essential oil formed a stable structure after absorbing the free radical lone pair of electrons, which can scavenge the free radicals generation in tomatoes cells [57]. There was no significant difference in MDA content between the two control groups throughout the storage period. On the 10^th^ day of storage, compared with CK1 and CK2, the MDA content of the optimal group was reduced by 6.00% and 7.84%. Therefore, KGM composite coating could inhibit the elevation of MDA content and reduce the degree of cell membrane lipid peroxidation and injury [58].

POD is an oxidoreductase enzyme commonly found in fruits and vegetables that catalyzes the decomposition of H_2_O_2_ into oxygen and water and does not cause damage to cells, so POD activity is used as an important indicator of whether systemic tissues can effectively scavenge free radicals [59–61]. The changes in POD activity of tomatoes were shown in Fig 8(f). The POD activity of each component of tomatoes showed a trend of increasing and then decreasing with the increase of storage time, and after the 4th cell destruction and senescence began to occur and the activity decreased. During the storage period, the POD activity of the two control groups was always lower than that of the optimal group, and there was always no significant difference in the POD activity of the two control groups during the storage period. On the 4^th^ day of storage, the highest value of POD activity appeared and there was a significant difference between the optimal group and both CK groups (*P* < 0.05), on the 10^th^ day of storage, the POD activity of the optimal group was extremely significantly higher than that of the CK1 and CK2 groups (*P* < 0.01) at 1.40 U/min, compared with CK1 and CK2, the POD activity of the optimal group increased by 29.63% and 25.00%. Although the POD activity of tomatoes showed a decreasing trend after day 4, the KGM composite coating treatment could maintain the POD activity of tomatoes at a relatively high level, which could better catalyze the decomposition of H_2_O_2_ in tomatoes and avoid peroxidative damage to tomatoes cells caused by excessive H_2_O_2_ accumulation [62–64].

### 3.5 Discussion

By studying the effects of cinnamon, clove, and mugwort essential oils on weight loss ratio and hardness of tomatoes and selecting essential oils and concentrations suitable for tomatoes preservation, the following discussion were made. Among the same types of essential oils, the concentration of 0.50 g/L cinnamon essential oil, 0.50 g/L clove essential oil and 1.00 g/L mugwort essential oil treated tomatoes were better for storage and preservation. What’s more, the best freshness preservation was achieved in tomatoes treated with 0.50 g/L cinnamon essential oil soaking, with a 10.87% and 9.85% reduction in weight loss and 8.37% and 10.69% increase in hardness compared to CK1 and CK2, respectively.

Then, the freshness preservation effects of cinnamon essential oil concentration, KGM concentration and soaking time on tomatoes were studied with weight loss ratio and hardness as evaluation indexes; and the best preservation effect of tomatoes was determined by single-factor analysis with KGM concentration of 9.00 g/L, soaking time of 3.00 min, and the concentration of cinnamon essential oil of 0.50 g/L. The best combination of freshness preservation conditions was cinnamon essential oil concentration of 0.70 g/L, KGM concentration of 8.20 g/L and soaking time of 3.20 min. The relative errors of weight loss ratio and hardness of tomatoes measured under this combination were small, 2.5% and 1.3%, respectively, which indicated that the response model could better predict the freshness preservation of tomatoes and verified the validity of the model [65].

Thus, 0.70g/L cinnamon essential oil and 8.20g/L KGM optimal composite coating preservative were prepared to study the storage and preservation effect of tomatoes at room temperature. The weight loss ratio of tomatoes in the optimal group (KGM compound coating group) was significantly decreased (*P* < 0.05), the hardness and TSS content of tomatoes in the optimal group were significantly increased (*P* < 0.01), which effectively delayed the softening of flesh tissue and kept the nutrition and flavor of tomatoes fruit. In addition, the optimal group could maintain the integrity of tomatoes cell membrane structure, effectively reduce the membrane permeability and MDA content of tomatoes, compared with CK1 and CK2; the Vc content in the optimal group was increased by 8.07% and 9.67%, and the POD activity was increased by 29.63% and 25.00%. The fresh-keeping effect is remarkable [66,67].

## 4 Conclusions

The work firstly concluded that the 0.50 g/L of cinnamon essential oil had the best preservation effects in terms of the size of evaluation indexes and material costs after comparing the effects of essential oils of cinnamon, clove and mugwort on the quality of tomatoes. The best preservation effect of tomatoes was determined by single factor analysis with KGM concentration of 9.00 g/L, soaking time of 3.00 min and the concentration of cinnamon essential oil of 0.50 g/L. The optimal combination of preservation conditions for the edible composite coating was determined to be 0.70 g/L cinnamon essential oil, 8.20 g/L konjac glucomannan (KGM), and an immersion time of 3.20 min. Under these conditions, the relative errors between the measured and predicted values for tomato weight loss and hardness were minimal, at 2.5% and 1.3%, respectively. This demonstrates that the response surface model effectively predicted tomato weight loss and hardness, validating its reliability. When applied to tomato preservation, the results indicated favorable efficacy. In conclusion, this study provides a theoretical foundation for the application of cinnamon essential oil-KGM composite coatings in tomato preservation. However, industrial-scale implementation requires addressing challenges such as essential oil stability and cost-effectiveness. Future research should focus on sustained-release carrier design and multi-variety compatibility validation to facilitate the transition of this technology from laboratory findings to sustainable supply chain applications.

## Supporting Information

S1 File This file contains Supplementary Figures. (DOCX)
