# Supplementary material for: Preservation effect of plant essential oil-KGM composite coating materials on the tomatoes
Source: PLoS One. 2025 Aug 18;20(8):e0328192. doi: 10.1371/journal.pone.0328192 (PMC12360612; doi:10.1371/journal.pone.0328192)
Supplement: S1 File — (DOCX) [file pone.0328192.s001.docx]

| Fig3(a)-Weight loss ratio/% | | | | | | | | | | | | |
| --- | --- | --- | --- | --- | --- | --- | --- | --- | --- | --- | --- | --- |
| Time | CK1 | error | CK2 | error | 3g/L | error | 6g/L | error | 9g/L | error | 12g/L | error |
| 0 | 0 | 0 | 0 | 0 | 0 | 0 | 0 | 0 | 0 | 0 | 0 | 0 |
| 2 | 0.217 | 0.07638 | 0.156 | 0.0506 | 0.246 | 0.023 | 0.189 | 0.00808 | 0.176 | 0.00656 | 0.163 | 0.03032 |
| 4 | 0.431 | 0.162 | 0.481 | 0.0185 | 0.447 | 0.05523 | 0.433 | 0.04232 | 0.395 | 0.016 | 0.431 | 0.016 |
| 6 | 0.945 | 0.09304 | 0.92033 | 0.07845 | 0.965 | 0.062 | 0.86967 | 0.01155 | 0.81167 | 0.03219 | 0.843 | 0.054 |
| 8 | 1.18233 | 0.02802 | 1.20567 | 0.07 | 1.22867 | 0.03153 | 1.15733 | 0.03885 | 1.114 | 0.04 | 1.222 | 0.023 |
| 10 | 1.485 | 0.03005 | 1.50533 | 0.02926 | 1.50433 | 0.04163 | 1.46467 | 0.03625 | 1.384 | 0.03727 | 1.431 | 0.022 |

| Fig3(b)-Hardness/N/cm2 | | | | | | | | | | | | |
| --- | --- | --- | --- | --- | --- | --- | --- | --- | --- | --- | --- | --- |
| Time | CK1 | error | CK2 | error | 3g/L | error | 6g/L | error | 9g/L | error | 12g/L | error |
| 0 | 0 | 0 | 0 | 0 | 0 | 0 | 0 | 0 | 0 | 0 | 0 | 0 |
| 2 | 0.217 | 0.07638 | 0.156 | 0.0506 | 0.1095 | 0.0015 | 0.1485 | 0.0095 | 0.102 | 0.006 | 0.1395 | 0.0065 |
| 4 | 0.431 | 0.162 | 0.481 | 0.0185 | 0.499 | 0.003 | 0.452 | 0.004 | 0.365 | 0.006 | 0.434 | 0.006 |
| 6 | 0.945 | 0.0885 | 0.92033 | 0.07845 | 0.923 | 0.03691 | 0.853 | 0.04454 | 0.78467 | 0.02991 | 0.77 | 0.02666 |
| 8 | 1.18233 | 0.02802 | 1.20567 | 0.07 | 1.156 | 0.04 | 1.09 | 0.01 | 1.041 | 0.003 | 1.083 | 0.012 |
| 10 | 1.485 | 0.03005 | 1.50533 | 0.02926 | 1.40233 | 0.02219 | 1.35533 | 0.04162 | 1.33267 | 0.03403 | 1.341 | 0.04678 |

| Fig3(c)-Weight loss ratio/% | | | | | | | | | | | | |
| --- | --- | --- | --- | --- | --- | --- | --- | --- | --- | --- | --- | --- |
| Time | CK1 | error | CK2 | error | 1min | error | 2min | error | 3min | error | 4min | error |
| 0 | 0 | 0 | 0 | 0 | 0 | 0 | 0 | 0 | 0 | 0 | 0 | 0 |
| 2 | 0.217 | 0.07638 | 0.156 | 0.0506 | 0.1095 | 0.0015 | 0.1485 | 0.0095 | 0.102 | 0.006 | 0.1395 | 0.0065 |
| 4 | 0.431 | 0.162 | 0.481 | 0.0185 | 0.499 | 0.003 | 0.452 | 0.004 | 0.365 | 0.006 | 0.434 | 0.006 |
| 6 | 0.945 | 0.0885 | 0.92033 | 0.07845 | 0.923 | 0.03691 | 0.853 | 0.04454 | 0.78467 | 0.02991 | 0.77 | 0.02666 |
| 8 | 1.18233 | 0.02802 | 1.20567 | 0.07 | 1.156 | 0.04 | 1.09 | 0.01 | 1.041 | 0.003 | 1.083 | 0.012 |
| 10 | 1.485 | 0.03005 | 1.50533 | 0.02926 | 1.40233 | 0.02219 | 1.35533 | 0.04162 | 1.33267 | 0.03403 | 1.341 | 0.04678 |

| Fig3(d)-Hardness/N/cm2 | | | | | | | | | | | | |  |
| --- | --- | --- | --- | --- | --- | --- | --- | --- | --- | --- | --- | --- | --- |
| Time | CK1 | error | CK2 | error | 1min | error | 2min | error | 3min | error | 4min | error |  |
| 0 | 30.267 | 0.30551 | 30.267 | 0.30551 | 30.267 | 0.30551 | 30.267 | 0.30551 | 30.267 | 0.30551 | 30.267 | 0.3055 | |
| 2 | 27.267 | 0.30551 | 27 | 0.6 | 28.267 | 0.41633 | 28.867 | 0.11547 | 28.6 | 0.6 | 28 | 0.2 | |
| 4 | 25.2 | 0.4 | 25 | 0.2 | 26.2 | 0 | 26.133 | 0.61101 | 26.533 | 0.11547 | 26.2 | 0.6 | |
| 6 | 21.8 | 0.2 | 21.53333 | 0.23094 | 22.2 | 0.4 | 22.6 | 0.72111 | 23.33333 | 0.57735 | 22.4 | 0 | |
| 8 | 19.13333 | 0.61101 | 19.46667 | 0.50332 | 20.6 | 0.6 | 21.26667 | 0.11547 | 21.6 | 0.2 | 21.1333 | 0.1155 | |
| 10 | 16.46667 | 0.50332 | 16.26667 | 0.41633 | 16.6 | 0.34641 | 16.93333 | 0.57735 | 17.93333 | 0.57735 | 17.1333 | 0.5033 | |

| Fig6(a)-Weight loss ratio/% | | | | | | |
| --- | --- | --- | --- | --- | --- | --- |
| Time | CK1 | error | CK2 | error | The best group | error |
| 0 | 0 | 0 | 0 | 0 | 0 | 0 |
| 2 | 0.172 | 0.05058 | 0.196 | 0.06586 | 0.171 | 0.02739 |
| 4 | 0.517 | 0.02848 | 0.547 | 0.02743 | 0.495 | 0.051 |
| 6 | 0.825 | 0.07427 | 0.841 | 0.02996 | 0.713 | 0.07921 |
| 8 | 1.125 | 0.11654 | 1.096 | 0.015 | 0.975 | 0.10121 |
| 10 | 1.357 | 0.064 | 1.327 | 0.08723 | 1.182 | 0.03467 |

| Fig6(b)-Hardness/N/cm2 | | | | | | |
| --- | --- | --- | --- | --- | --- | --- |
| Time | CK1 | error | CK2 | error | The best group | error |
| 0 | 32 | 0.52915 | 32 | 0.52915 | 32 | 0.52915 |
| 2 | 30.73333 | 0.11547 | 30.46667 | 0.231 | 31.73333 | 0.57735 |
| 4 | 27.46667 | 0.64291 | 27.8 | 1.11355 | 29.6 | 0.72111 |
| 6 | 22.66667 | 0.98658 | 23.2 | 1 | 25.46667 | 1.02632 |
| 8 | 19.8 | 0.8 | 20.2 | 1.058 | 23.26667 | 1.28582 |
| 10 | 16 | 1.0583 | 15.66667 | 0.80829 | 19.33333 | 0.70238 |

| Fig6(c)-TSS content/% | | | | | | |
| --- | --- | --- | --- | --- | --- | --- |
| Time | CK1 | error | CK2 | error | The best group | error |
| 0 | 3.36667 | 0.05774 | 3.36667 | 0.05774 | 3.36667 | 0.05774 |
| 2 | 3.46667 | 0.05774 | 3.43333 | 0.05774 | 3.33333 | 0.05774 |
| 4 | 3.5 | 0.1 | 3.53333 | 0.05774 | 3.43333 | 0.05774 |
| 6 | 3.13333 | 0.05774 | 3.2 | 0.1 | 3.6 | 0.1 |
| 8 | 2.93333 | 0.15275 | 2.96667 | 0.05774 | 3.367 | 0.058 |
| 10 | 2.9 | 0.1 | 2.86667 | 0.11547 | 3.267 | 0.05774 |

| Fig6(d)-Vc content/mg/100g | | | | | | |
| --- | --- | --- | --- | --- | --- | --- |
| Time | CK1 | error | CK2 | error | The best group | error |
| 0 | 20.65 | 0.16829 | 20.65 | 0.16829 | 20.65 | 0.16829 |
| 2 | 20.894 | 0.11172 | 20.8445 | 0.12374 | 21.2765 | 0.39386 |
| 4 | 21.9975 | 0.4278 | 21.7595 | 0.38537 | 22.7565 | 0.378 |
| 6 | 20.534 | 0.22769 | 20.8845 | 0.18031 | 21.2155 | 0.216 |
| 8 | 20.157 | 0.14001 | 20.187 | 0.26587 | 21.0175 | 0.2284 |
| 10 | 19.198 | 0.195 | 18.917 | 0.322 | 20.75433 | 0.33496 |

| Fig6(e)-POD activity/U/min·g | | | | | | |
| --- | --- | --- | --- | --- | --- | --- |
| Time | CK1 | error | CK2 | error | The best group | error |
| 0 | 1.4015 | 0.31325 | 1.4015 | 0.31325 | 1.4015 | 0.31325 |
| 2 | 1.482 | 0.184 | 1.381 | 0.121 | 1.653 | 0.268 |
| 4 | 2.199 | 0.127 | 1.977 | 0.222 | 2.61 | 0.16 |
| 6 | 1.736 | 0.254 | 1.794 | 0.141 | 2.046 | 0.11 |
| 8 | 1.177 | 0.203 | 1.082 | 0.067 | 1.305 | 0.075 |
| 10 | 1.078 | 0.05718 | 1.123 | 0.019 | 1.398 | 0.01 |

| Fig6(f)-MDA content/mmol/g Fw | | | | | | |
| --- | --- | --- | --- | --- | --- | --- |
| Time | CK1 | error | CK2 | error | The best group | error |
| 0 | 0.14567 | 0.0235 | 0.14567 | 0.0235 | 0.14567 | 0.0235 |
| 2 | 0.249 | 0.019 | 0.26967 | 0.0215 | 0.208 | 0.019 |
| 4 | 0.348 | 0.021 | 0.359 | 0.03 | 0.28067 | 0.0455 |
| 6 | 0.434 | 0.034 | 0.381 | 0.029 | 0.37367 | 0.0145 |
| 8 | 0.44633 | 0.017 | 0.46133 | 0.0215 | 0.405 | 0.025 |
| 10 | 0.494 | 0.006 | 0.50133 | 0.012 | 0.474 | 0.006 |
